# Dose prescription for stereotactic body radiotherapy: general and organ-specific consensus statement from the DEGRO/DGMP Working Group Stereotactic Radiotherapy and Radiosurgery

**DOI:** 10.1007/s00066-024-02254-2

**Published:** 2024-07-12

**Authors:** Thomas B. Brunner, Judit Boda-Heggemann, Daniel Bürgy, Stefanie Corradini, Ute Karin Dieckmann, Ahmed Gawish, Sabine Gerum, Eleni Gkika, Maximilian Grohmann, Juliane Hörner-Rieber, Simon Kirste, Rainer J. Klement, Christos Moustakis, Ursula Nestle, Maximilian Niyazi, Alexander Rühle, Stephanie-Tanadini Lang, Peter Winkler, Brigitte Zurl, Andrea Wittig-Sauerwein, Oliver Blanck

**Affiliations:** 1 Department of Radiation Oncology, Medical University of Graz, Auenbruggerplatz 32, 8036 Graz, Austria; 2 Department of Radiation Oncology, University Medicine Mannheim, Medical Faculty Mannheim, Heidelberg University, Mannheim, Germany; 3 Department of Radiation Oncology, University Hospital, LMU Munich, Munich, Germany; 4 Department of Radiotherapy, University Medical Center Giessen-Marburg, Marburg, Germany; 5 Department of Radiation Oncology, Paracelsus University Salzburg, Salzburg, Austria; 6 Department of Radiation Oncology, University Hospital Bonn, 53127 Bonn, Germany; 7 Department of Radiotherapy and Radiation Oncology, University Medical Center Hamburg-Eppendorf, Martinistr. 52, 20246 Hamburg, Germany; 8 Department of Radiation Oncology, Heidelberg University Hospital, Im Neuenheimer Feld 400, 69120 Heidelberg, Germany; 9 Department of Radiation Oncology, Medical Center-University of Freiburg, Faculty of Medicine, Freiburg, Germany; 10 Department of Radiotherapy and Radiation Oncology, Leopoldina Hospital Schweinfurt, Robert-Koch-Straße 10, 97422 Schweinfurt, Germany; 11 Department of Radiation Oncology, University Hospital Leipzig, Stephanstraße 9a, 04103 Leipzig, Germany; 12 Department of Radiation Oncology, Kliniken Maria Hilf, Moenchengladbach, Germany; 13 Department of Radiation Oncology, Eberhard Karls University Tübingen, Tübingen, Germany; 14 Department of Radiation Oncology, University Hospital Zurich, University of Zurich, Rämistrasse 100, 8091 Zurich, Switzerland; 15 Department of Therapeutic Radiology and Oncology, Comprehensive Cancer Center, Medical University of Graz, 8036 Graz, Austria; 16 Department of Radiation Oncology, University of Würzburg, Würzburg, Germany; 17 Department of Radiation Oncology, University Medical Center Schleswig-Holstein, Arnold-Heller-Straße 3, 24105 Kiel, Germany

**Keywords:** Consensus, Radiation oncology, Stereotactic body radiotherapy, Dose prescription, ICRU report 91

## Abstract

**Purpose and objective:**

To develop expert consensus statements on multiparametric dose prescriptions for stereotactic body radiotherapy (SBRT) aligning with ICRU report 91. These statements serve as a foundational step towards harmonizing current SBRT practices and refining dose prescription and documentation requirements for clinical trial designs.

**Materials and methods:**

Based on the results of a literature review by the working group, a two-tier Delphi consensus process was conducted among 24 physicians and physics experts from three European countries. The degree of consensus was predefined for overarching (OA) and organ-specific (OS) statements (≥ 80%, 60–79%, < 60% for high, intermediate, and poor consensus, respectively). Post-first round statements were refined in a live discussion for the second round of the Delphi process.

**Results:**

Experts consented on a total of 14 OA and 17 OS statements regarding SBRT of primary and secondary lung, liver, pancreatic, adrenal, and kidney tumors regarding dose prescription, target coverage, and organ at risk dose limitations. Degree of consent was ≥ 80% in 79% and 41% of OA and OS statements, respectively, with higher consensus for lung compared to the upper abdomen. In round 2, the degree of consent was ≥ 80 to 100% for OA and 88% in OS statements. No consensus was reached for dose escalation to liver metastases after chemotherapy (47%) or single-fraction SBRT for kidney primaries (13%). In round 2, no statement had 60–79% consensus.

**Conclusion:**

In 29 of 31 statements a high consensus was achieved after a two-tier Delphi process and one statement (kidney) was clearly refused. The Delphi process was able to achieve a high degree of consensus for SBRT dose prescription. In summary, clear recommendations for both OA and OS could be defined. This contributes significantly to harmonization of SBRT practice and facilitates dose prescription and reporting in clinical trials investigating SBRT.

**Supplementary Information:**

The online version of this article (10.1007/s00066-024-02254-2) contains supplementary material, which is available to authorized users.

## Introduction

More than 5 years ago, the International Commission on Radiation Units and Measurements (ICRU) report 91 on prescribing, recording, and reporting of stereotactic treatments with small photon beams was published [1]. For the first time, specific recommendations were available for a radiotherapy technique that had long made the transition from pioneering centers to the vast majority of departments and institutions of radiation oncology, as reflected in the exponential rise of publications on stereotactic radiotherapy over the past decades: 94 in 1990 to 2487 in 2020 (Suppl. Figure 1). Primarily, the ICRU report 91 recommends prescription of the dose to the isodose surface that covers an optimal percentage of the planning target volume (PTV) while optimally restricting the dose to organs at risk (OAR). To better understand the practical consequences of this, several recommendations and commentaries from the working groups on stereotactic radiotherapy and radiosurgery of the German Radiation Oncology and Medical Physics Societies (DEGRO/DGMP) were published, putting the report 91 into clinical context [2–4].

However, to date, many centers are still struggling with the implementation of ICRU report 91, especially with respect to the comprehensive set of recommended target coverage and dose metrics as well as OAR dose limitations [1, 5]. This struggle is even more pronounced for extracranial indications, where patient and target motion as well as dose calculation and treatment delivery uncertainties can significantly affect dose prescription. This problem stimulated a number of multicentric, multiplatform benchmark planning studies within the DEGRO/DGMP working groups, where selected cases of stereotactic body radiotherapy (SBRT) were distributed among experienced centers to analyze the comparability of the resulting treatment plans of lung, liver, and pancreatic targets [6–9]. Of note, the conception and implementation of these planning studies was interdisciplinary, including experienced physicians and medical physicists; however, the ultimate responsibility for plan acceptance lies with the radiation oncologist. Hence, physicians will eventually need to be capable of accurately defining multiparametric dose prescription concepts for stereotactic radiotherapy prior to initiation of the treatment planning process. This leaves no room for aficionados of the famous R&B song lines “I don’t need no doctor for my prescription to be made” in SBRT [10].

To give a more practical example, the need for harmonization of treatment planning and dose prescription, which go hand in hand, is illustrated by the comparison of two peripheral lung SBRT benchmarks from our working groups [6, 7]. In a planning study with insufficient definition of dose metrics, significant variations in mean PTV dose and dose conformity indices were reported, underlining the need for further harmonization and standardization [6]. In the second planning study, where additional dose prescription parameters were used—specifically the addition of median/mean dose prescription values—the treatment plans were significantly more harmonized as compared to the first study [7]. However, not only more consistent plans among different centers and techniques but also better prediction of local control is expected with improved multiparametric dose prescription concepts, because local tumor control seems to correlate better and independently with median compared with near-minimal doses of the target volumes [11, 12].

Therefore, on top of previously published organ-specific reports, our working groups aim to provide [13–15] recommendations on multiparametric dose prescription and reporting for SBRT in relation to ICRU report 91 in addition to our existing quality requirements for stereotactic radiotherapy [3, 4] to guide current clinical practice and the design of future clinical trials. In our current work, experts in stereotactic radiotherapy systematically summarized literature published by our working groups and included important publications on the topic by other renowned groups to establish a consensus on evidence-based strategies and to prescribe doses effectively and safely for SBRT treatments.

## Methods

We reached out to radiation oncologists and medical physicists specializing in radiation oncology from Germany, Austria, and Switzerland. Specifically, we targeted individuals who demonstrated significant involvement in the DEGRO/DGMP working groups on stereotactic radiotherapy and radiosurgery, and who were recognized as experts in the field based on their professional profiles, publication records, and academic collaborations. Twenty-seven invitations were made and 24 agreed to participate in the project. Due to a general paucity of available published data on SBRT conforming with the ICRU report 91, a systematic literature review in accordance with the Preferred Reporting Items for Systematic Reviews and Meta-Analyses (PRISMA) criteria was deemed unfeasible. Therefore, we systematically summarized literature published within our working groups and added relevant International Commission on Radiation Units and Measurements (ICRU) report 91-conform publications of other renowned groups. We then chose a modified Delphi consensus approach to develop specific statements for dose prescriptions for SBRT treatments.

The modified Delphi technique employing online questionnaires as a form of a structured, transparent, and iterative approach was formulated based on the literature as described above. During the process, we obtained anonymous feedback and allowed participants to reassess their own judgements based on feedback in discussions [16, 17]. A web-based survey platform was used (Google Forms, Meta, Menlo Park, USA). The survey was performed blinded to participant responses during each round and two rounds took place.

Prior to the first round, statements on dose prescription in SBRT of both overarching and organ-specific nature were developed and phrased in weekly video conferences from July to October 2023. Prior to voting, thresholds for high, intermediate, and poor agreement were set at ≥ 80%, 60–79.9%, and < 60%, similar to other Delphi consensus studies [18]. In the first round, voting allowed the three options “yes,” “no,” and “abstention.” The results were analyzed and presented in a videoconference after the voting process. Additionally, on November 25, 2023, the results were presented and discussed openly at a meeting of the working groups with 125 participants.

After the meeting and prior to the second round, statements were refined where necessary, to better measure the opinion of the participants. In the second round, statements were presented alongside summary data from the first round, and we modified the voting options to “yes,” “no,” and “not sufficiently qualified.” Note that abstention was removed as an option to obtain definitive answers on agreement. Subsequently, the results were analyzed and discussed in a final expert meeting under the consideration of up-to-date relevant published evidence on multiparametric dose prescription concepts.

## Results

In the first round, overarching and site-specific recommendations were voted on anonymously (supplementary tables 1 and 2). The results of 14 overarching statements demonstrated high agreement in 11 (79%) and moderate agreement in 3 of the 14 statements (21%). The three statements with moderate agreement were from three different thematic blocks (dose prescription, beam technique planning, and dose calculation). Agreement for organ-specific statements was significantly worse in the first round (seven statements with high and moderate and six with poor agreement). Statements concerning SBRT of primary and secondary liver lesions (seven statements) had the poorest agreement (57%) and only one statement achieved high agreement. Non-hepatic statements with poor agreement concerned lung and renal cell cancer in one instance each. In general, the option for abstention ranged from 14 to 36% in statements with poor agreement, and this was identified as an obstacle to sufficient interpretation of the agreement on according statements.

During a face-to-face meeting of members of the DEGRO/DGMP working groups in late November 2023, the results of the first round were presented and discussed in the plenum. Consequently, the group of experts decided to modify the process for the second round of voting. Available voting options were then “yes,” “no,” and “not sufficiently qualified,” e.g., if physicians did not feel qualified to vote on a physics statement and vice versa, and “abstention” was removed as option. Furthermore, several statements were semantically reviewed to better specify the questions.

**Fig. 1:**
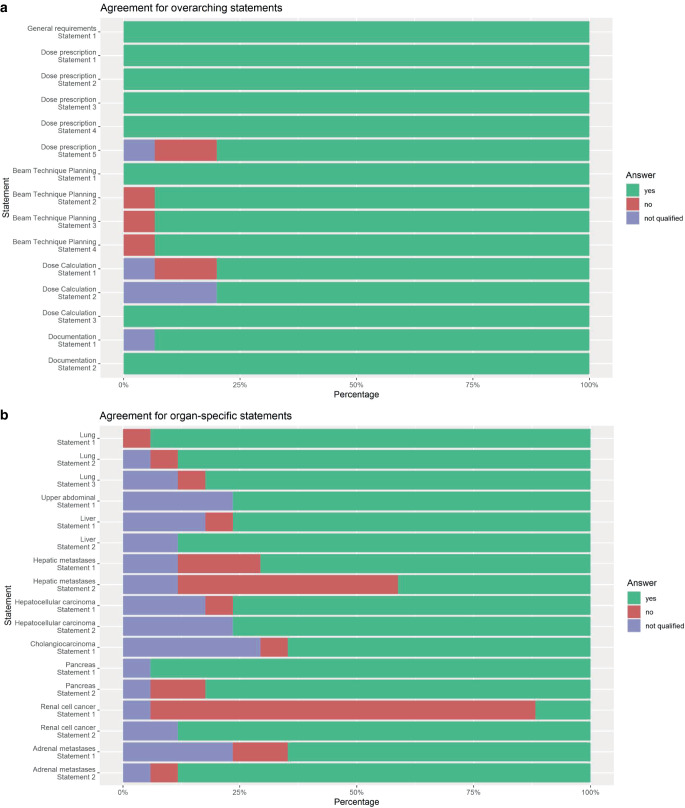
Percentage of agreement in the second round of the Delphi process for each of the statements with the options to vote yes, no, or not qualified. Not qualified describes statements for which either physicians or physicists did not rank the statement to be part of their expertise. **a** Agreement rates for overarching statements; **b** agreement rates for organ-specific statements

In December 2023, the second round of anonymous voting within the group of experts was conducted. The results of this second round are presented in Fig. 1 and Tables 1 and 2. In the second round, all 14 overarching questions achieved high agreement (mean 96.6%, median 100%, range 86–100%). For the organ-specific questions, 15 achieved high and 2 poor agreement (mean 86.2%, median 93%, range 13–100%). Specifically, no agreement was found concerning liver metastases, where differential dose prescription as a function of prior chemotherapy was refuted and radiosurgery (i.e., single-fraction SBRT) was rejected for renal cell cancer as standard of care for this indication.

**Table 1:** Expert consensus on overarching statements of dose prescription in stereotactic body radiotherapy in round 2 (votes of 17 experts)

Topic	Question	Percentage of agreement
Dose prescription	The dose for stereotactic radiotherapy treatments with small photon beams should be prescribed to a PTV-encompassing isodose (e.g., PTV D95–98%) and reported based on the recommendations of ICRU report 91	100
	The desired dose prescription based on a PTV-encompassing isodose must be accompanied by further dosimetric goals to be achieved in the GTV (e.g., GTV D95–98%, GTV D50%, GTV D2%)	100
	For treatment plan harmonization (e.g., within clinical trials), a dose prescription to the GTV median dose (GTV D50%) could be considered if further dosimetric goals for the PTV are provided (e.g., PTV D95–98%, PTV D2%)	100
	The linear quadratic model should be used to convert different SBRT dose prescription schemes from one to another for 3 or more fractions	86
Beam technique planning	Best practice guidelines including simultaneous integrated protection and boost concepts must be considered to achieve optimal trade-offs between target dose coverage and critical structure sparing for SBRT treatment planning	100
	For type I re-SBRT after prior SBRT, dose accumulation is strongly advised	94
	For type I re-SBRT after prior SBRT, appropriate image registration methods are required	94
	To create robust SBRT treatment plans, the reduction of interplay effects should be considered (e.g., through monitoring and reduction of uncompensated motion and/or high beam modulation)	94
Dose calculation	Density override in the treatment planning systems of artifacts in or near the PTV ideally after the use of metal artifact reduction (MAR) algorithms should be considered for accurate dose calculation for stereotactic radiotherapy treatments	88
	In areas with large density inhomogeneities, the use of a dose calculation algorithm that considers lateral electron transport to correct for density inhomogeneities is required for stereotactic radiotherapy treatments	100
	The maximum grid size for dose calculation for stereotactic radiotherapy treatments should be 1–2 mm according to the target lesion dimensions and the image resolution for target definition	100
Documentation	The dosimetric plan information of stereotactic radiotherapy treatments with small photon beams must be documented and reported according to ICRU report 91 with, at minimum, *the PTV prescription dose,* *D* *(PTV)*_*near-min*_^*ab*^*,* *D* *(PTV)*_*50%*_ *D* *(PTV)*_*near-max*_^*a*^ *D* *(GTV)*_*50%*_* and D* *(GTV)*_*near-max*_^*a*^ *if applicable D* *(ITV)*_*50%*_* and D* *(ITV)*_*near-max*_^*a*^ *all OARs: D*_*near-max*_* and D*_*mean*_	100
	Due to the potential of significant dose reductions from uncompensated residual target motion during stereotactic radiotherapy treatments, the motion management strategy must be reported in alignment with ICRU report 91	100

*D* dose, *GTV* gross target volume, *ICRU* International Commission on Radiation Units and Measurements, *ITV* internal target volume, *Max* maximal, *Min* minimal, *OAR* organ at risk, *PTV* planning target volume, *SBRT* stereotactic body radiotherapy

^a^Except for PTVs ≤ 2 ml: (1) for PTV ≤ 2 ml, near-min is an absolute volume of 0.035 ml (i.e., 0.035 cm^3^), in which case DV‑0.035 ml is reported (DV-0.035 ml = the dose to the total volume V of a region of interest minus 0.035 ml); (2) for a PTV ≤ 2 ml, near-max is an absolute volume of 0.035 ml, in which case D0.035 ml is reported

^b^Acccording to ICRU report 91, *D*_*near-min*_ is *D*_*98%*_; however for lung lesions *D*_*95%*_ is recommended by the authors due to the impact of air surrounding the GTV on dose deposition

**Table 2:** Expert consensus on organ-specific statements of dose prescription in stereotactic body radiotherapy in round 2 (votes of 17 experts)

Topic	Question	Percentage of agreement
Lung	For peripheral tumors the dose should be escalated to the GTV/ITV while ensuring a reasonable dose to the PTV	94
	For ultracentral tumors, dose sparing of the bronchial tree, trachea, and esophagus based on clinically accepted dose limitations must have priority over PTV coverage. (1^a^)	94
	Dose prescription to primary lung tumors should not be different than for pulmonary metastases if ablation is intended [11] (2^a^)	93
Upper abdomen	Dose prescription to upper abdominal target volumes that are close to stomach and/or duodenum should imply the administration of prophylactic gastric acid reduction with proton pump inhibitors. (4^a^)	100
Liver in general	Dose prescription to PTV areas containing patent, i.e., non-tumor affected/non-obstructed central bile ducts, specifically the common bile duct and the common hepatic duct, should avoid dose escalation in these areas [19–21] (3^a^)	93
	Liver function must be taken into account for dose prescription, especially in HCC (protected liver volume, Child–Pugh score, ALBI score, etc.), to avoid the risk of radiation-induced liver disease. (2^a^)	100
Hepatic metastases	Liver metastases from colorectal cancer should be treated with higher prescription dose compared to liver metastases from other primary tumors to achieve similar local control [22] (2^a^)	80
	Liver metastases should be treated with higher prescription doses after chemotherapy compared to chemotherapy-naive liver metastases [22] (2^a^)	47
Hepatocellular carcinoma	Since a clinically accepted dose–response relationship (tumor control probability, TCP) is not known, dose prescription should be adapted to liver function to reduce the risk of radiation-induced liver disease. (3^a^)	93
	Dose prescription in patients with Child–Pugh scores > 8 should maximize sparing of non-tumor liver volume including reduction of the prescribed dose to target volumes in an individualized approach [23]. (4^a^)	100
Cholangiocarcinoma	Dose prescription for primary intrahepatic and perihilar cholangiocarcinoma should not be different than for liver metastases. (5^a^)	92
Pancreatic ductal adenocarcinoma	Pancreatic lesions should be treated with 5 or more fractions outside of prospective clinical trials. (1^a^)	100
	For pancreatic lesions, dose sparing of hollow OARs based on clinically accepted dose limitations must have priority over PTV coverage. (1^a^)	88
Renal cell cancer	Renal cell cancer should be treated with single-fraction SBRT due to radiobiological characteristics of the tumors [24, 25]. (1^a^)	13
	Dose prescription should be based on the results of a split renal function to determine the relative contribution of each of the two kidneys to total renal function, e.g., with renal scintigraphy. (2^a^)	100
Adrenal metastases	Dose prescription for both bilateral and unilateral adrenal SBRT should mandate hormone and/or endocrinological monitoring in the planning process [26, 27]. (4^a^)	85
	Dose prescription should prioritize tolerance doses of neighboring organs at risk (e.g., stomach, duodenum, small bowel) over PTV coverage. (1^a^)	94

*ALBI* albumin-bilirubin score, *GTV* gross target volume, *HCC* hepatocellular carcinoma, *ITV* internal target volume, *OAR* organ at risk, *PTV* planning target volume, *SBRT* stereotactic body radiotherapy

^a^number of votes “insufficiently qualified”

## Discussion

To our knowledge, this is the first international recommendation for multiparametric dose prescription in SBRT considering ICRU report 91. Literature screening and a detailed summary of published manuscripts from our working groups prior to the Delphi process showed that the body of evidence on the topic is still limited and requires further research and consequent reporting of ICRU report 91-conform metrics. Nevertheless, it was possible to develop a consensus on overarching and organ-specific statements in the sense of a least common denominator which provides guidance and will allow addition of further elements.

### Overarching—dose prescription

The ICRU report 91 was a valuable first step toward closing the gap between a large rise in stereotactic radiotherapy treatments and the need for harmonization of dose prescription [1, 2]. Earlier ICRU reports prescribed to the ICRU point (ICRU 50) and later to the median dose (ICRU 83), all under the assumption of homogenous dose distributions. A major impact of ICRU report 91 was the recommendation that for stereotactic radiotherapy treatments, the dose should be prescribed as an isodose surface that covers an optimal percentage volume of the PTV. However, the question remains of whether that alone is sufficient to fully specify required stereotactic radiotherapy dose distributions and reflect emerging evidence of clinical outcome.

Moving forward, this Delphi process achieved absolute consensus on the requirement of additional dosimetric goals beside the prescription isodose and PTV coverage, e.g., for the gross target volume (GTV). The literature specifically addressing these objectives is sparse, yet insights can be derived from the outcomes of existing studies, particularly those emphasizing the median dose to the GTV as pivotal for local control and the harmonization of treatment protocols. [7–9, 12]. Nevertheless, future studies changing the primary prescription priority from near-minimal PTV dose to median GTV dose are necessary to test this hypothesis for further improved prediction of local control.

In addition to dose prescription and the requirement to harmonize the desired dose inhomogeneity, clinical factors necessitate adjustments in dose prescription, leading to the adaption of fractionation schemes. However, this creates another complex problem regarding the comparability of the biological value of dose for both targets and OARs. The linear quadratic model achieved high consensus in this Delphi process for being suitable for such comparisons based on analyses from our working groups when restricting it to ≥ 3 fractions [22, 28]. While the biologically equivalent dose (BED) represents a calculation model to quantify the effective dose to target lesions by integrating the physical dose with its biological effects, clinicians prefer equivalent doses in 2‑Gy fractions (EQD2) to estimate the safety of a treatment plan for OARs, since they correspond better to clinical experience from conventional fractionation [29].

#### Beam technique planning

Multiparametric dose prescription and BED/EQD2 estimation then directly translate into the beam technique planning. While stereotactic radiotherapy in general demands highly accurate dose delivery, the actual accuracy of SBRT depends on patient setup accuracy and image guidance as well as the motion management strategy and the technical standard of the irradiation device [4, 30], all of which eventually influence the multiparametric dose prescription. In other words, the more accurate the treatment delivery, the more accurate the dose prescription can be during treatment planning.

Measures to increase the treatment accuracy for SBRT are well known. Immobilization may be achieved with stereotactic body frames or specialized immobilization equipment (e.g., vacuum cushion) in combination with image-guidance technology [31, 32]. Motion management for treating moving tumors with SBRT is crucial and information on the extent of motion is vital to assign appropriate safety margins. To assess motion prior to dose optimization, planning imaging at specific breathing phases or 4D CT are also becoming integrated into routine practice as well as means to reduce motion such as abdominal compression, gating, or tracking techniques [33, 34].

However, irrespective of passive or active motion management strategies, safety margins, and treatment uncertainties, this Delphi consensus highly agrees to consider all the aforementioned for dose prescription and reporting according to the ICRU report 91. While in clinical practice we can achieve highly conformal treatment plans with rapid dose fall-offs through intensity-modulation techniques [34–39], the dose prescription to a gating or tracking plan may look very different to an internal target volume (ITV) plan in which larger portions of healthy tissue may be included in the PTV. Adding to that, respiratory motion may introduce interplay effects reducing the dose prescription accuracy further, and strategies to reduce these effects are strongly recommended [39, 40].

A complex challenge, even with multiparametric dose prescription, is the integration of simultaneous boost and protection concepts (SIB/SIP) to enhance the safe delivery of high biological doses to tumor volumes while optimally restricting dose to close critical organs or their planning risk volumes (PRVs). An intersection volume between a PRV and the PTV defines the protection volume (PTV_SIP) in which the prescribed dose is only reduced to such an extent that dose constraints for that OAR can be sufficiently maintained [41, 42]. A high level of consensus was achieved in this work on the integration of these techniques into treatment planning, and the SIB concepts can be readily implemented with a GTV mean dose prescription. However, the SIP concept inevitably translates into reduced PTV coverage, which cannot easily be captured by the proposed dose prescription metric in ICRU report 91. Further prescription metrics are required and must be considered for SBRT close to radiation-sensitive OARs. To complicate the matter further, re-irradiation will also influence the multiparametric dose prescription, and registration of previous treatment plans as well as dose accumulation should be considered accordingly [43–46].

#### Dose calculation

Starting from beam technique planning, the basis for dose prescription is of course dose calculation, and several aspects can lead to deviations and inaccuracies in the calculated dose that cannot be neglected from a clinical perspective. Especially for small targets and for treatment plans exhibiting steep dose gradients, the dose calculation accuracy will influence the dose–volume parameters of the target volumes and, consequently, the prescribed dose.

There is sufficient consensus by now that type‑a (or factor-based) dose calculation algorithms are not adequate and should not be used in SBRT for thoracic and abdominal targets [1, 4, 47]. Particularly for lung SBRT, correct modelling of lateral electron transport is essential to ensure accurate calculation in heterogeneous media. It has been shown that volume–dose indices depend on the calculation algorithm [48, 49], leading to systematic overestimations of target volume doses in heterogeneous media, even when type‑b algorithms are used as compared to type‑c algorithms (Monte Carlo or LBTE-solver algorithms) [50–52]. This effect might be even more significant in deep-inspiration breath-hold (DIBH) techniques [50–53]. The use of type‑b algorithms for SBRT is a minimum requirement; however, for lung SBRT and in areas exhibiting distinct tissue density heterogeneities, the use of type‑c algorithms is strongly recommended. Especially in lung target volumes, the dose should be preferably calculated to medium rather than to water, since systematic “shifts” have to be considered when type‑c algorithms are compared to type‑b algorithms [54].

The resolution of the calculation grid also affects the accuracy of the dose calculation and consequently it influences the dose–volume parameters which we prescribe to. Literature about the magnitude of this effect for SBRT is sparse [55, 56], but in agreement with published guidelines, a grid resolution of smaller than or equal to 2 mm is recommended [1, 4]. For very small targets (< 2 ccm), a dose grid resolution of 1 to 1.5 mm should be considered.

Lastly, the effect of image artifacts in the vicinity of metallic implants on dose calculation has been investigated mostly for treatments in the head and neck, pelvic, and spinal regions [57, 58]. It must be assumed that quantitatively comparable dose deviations exist in SBRT. Metal artifact reduction algorithms should be considered for CT reconstruction to minimize the implications for dose prescription. The areas with obviously remaining artifacts should be segmented and the density should be overridden to water or the most likely local tissue density. .

#### Documentation

Precise and comprehensive reporting of the prescribed and planned dose is essential for stereotactic radiotherapy treatments. For dose reporting, the definitions given in ICRU report 91 must be followed. As a minimum requirement, reporting of the dose prescribed to the PTV, D (PTV)_98%_, D (PTV)_50%_, D (PTV)_near-min_, D (GTV)_50%_, D (GTV)_near-max_, and, if applicable, D (ITV)_50%_ and D (ITV)_near-max_, is obligatory. Furthermore, D_near-max_ and D_median_ must be reported for all relevant OARs. Due to the potential of significant dose reductions from uncompensated residual target motion during stereotactic radiotherapy treatments, the motion management strategy must also be reported in alignment with ICRU report 91.

### Organ-specific

#### Lung

Moving on from overarching statements to site-specific statements on dose prescription, the lung is undoubtedly the most researched organ for SBRT [6]. Based on data of a modeling study including 1500 cases of pulmonary SBRT, PTV BED_average_ (the average between BED_max_ and BED_min_ of the PTV) generally correlated better with tumor control probability than either PTV BED_max_ or PTV BED_min_ [12, 59]. Importantly, GTV D_mean_ was closely correlated with PTV BED_average_ in the analysis by Klement and coauthors [12]. Hence, more emphasis should be placed on achieving sufficiently high mean doses within the GTV for peripheral lung tumors while ensuring a reasonable dose to the PTV [7].

Ultracentral tumors are commonly defined as tumors in which the PTV overlaps with the proximal bronchial tree or the esophagus. Concerns over severe complications following SBRT of ultracentral tumors were raised after significantly increased grade 3 toxicity (including fatal pulmonary hemorrhage) was reported in up to 40% of patients [60]. The HILUS trial, which treated 65 patients with ultracentral lung tumors with 8 × 7 Gy with a maximum dose up to 150% of the prescribed D_near-min_, achieved good local control rates but at the expense of highly increased grade 3 to 5 toxicity in 22 patients, including 10 cases of treatment-related deaths [61]. In contrast, others did not detect significant differences in high-grade toxicity when comparing pulmonary SBRT for ultracentral to central tumors [62]. However, toxicity after SBRT to central and ultracentral tumors is significantly higher as compared to peripheral lesions. Furthermore, there is an inherent risk of bleeding by tumors bridging central bronchi and large vessels, which adds risk beyond radiation-induced toxicity. Both should be discussed with the patient versus less hypofractionated radiotherapy or radiochemotherapy for decision making.

Therefore, this Delphi consensus agrees that OAR sparing based on clinically accepted dose limitations should have priority over PTV coverage for central and ultracentral lung tumors [63]. To maximize the potential for safe dose escalation in pulmonary central SBRT, the maximum dose in the PTV may be limited as done in the EORTC LungTech trial (8 × 7.5 Gy to the median in the PTV with maximum dose limit at 120% of the prescription dose) [64, 65]. Running clinical studies like the SUNSET trial may help to refine criteria for patient selection in the future; however, in the SUNSET trial, tumors with endobronchial involvement were excluded [63, 66]. Modern high-precision image-guided radiation techniques such as MR-guided adaptive RT may be also used to achieve safer dose escalation. Prospective trials evaluating the benefit of adaptive MRgRT are recruiting (STAR-LUNG STUDY [NCT05354596] and MAGELLAN [NCT04925583]). Outside prospective clinical studies, ultracentral lung tumors, especially with endobronchial infiltration, should not be treated with aggressive hypofractionation.

Lastly, different histologies may theoretically exhibit a different radiosensitivity to primary non-small-cell lung cancer (NSCLC); however, no significant differences in dose–response relationships have been found so far for SBRT [11, 67, 68]. Therefore, the optimal dose fractionation schedule for lung metastases is primarily extrapolated from prospective data in early-stage NSCLC [69]. However, current on-going trials prescribe lower doses for ablative SBRT in pulmonary oligometastasis or progression.

#### Liver

Conversely, specific dose prescription depending on the underlying histology of secondary tumors, the presence of a cholangiocarcinoma, or the prior administration of chemotherapy was strongly suggested as a parameter impacting on local control in an analysis of SBRT of 452 liver lesions [22, 70]. The importance of high doses to achieve a high tumor control probability was found for colorectal cancer and for metastases pretreated with chemotherapy. The expert panel confirmed the influence of colorectal cancer histology in this Delphi process, but disagreed on prior chemotherapy impacting dose prescription, the latter most likely due to the clinical complexity which cannot be addressed by a binary function of prior chemotherapy administration [22, 70–72].

To balance adequate dose to the tumor versus toxicity, the most important life-threatening dose-limiting factors for liver SBRT are serial gastrointestinal OARs (stomach, esophagus, duodenum, and bowel with possible ulceration; bleeding and perforation), especially if directly in contact with the GTV (typically in segments II–III). In addition to appropriate motion management strategies and tight margins, multiparametric dose prescription should strictly respect the dose constraints of these organs and, if necessary, limit the PTV dose accordingly [19, 20]. Furthermore, dose prescription for liver lesions is often limited by the dose constraints of the liver itself and the risk of radiation-induced liver disease (RILD) [14, 23, 73]. This is especially true for hepatocellular carcinomas (HCC) in patients with liver cirrhosis, where fatal RILD has already been observed after SBRT and can only be avoided by consistent use of dose constraints [74]. In this context, liver function (Child–Pugh/ALBI score) must be taken into account when prescribing the dose for liver SBRT [75, 76]. For CP score ≥ 8, SBRT should only be considered with caution in view of the limited safety evidence [5–7, 9, 74]. Furthermore, in case of large metastases or also after major liver surgery, dose constraints must be strictly adhered to to avoid RILD [14, 19, 20, 77, 78].

As a different aspect of liver SBRT, dose constraints for central hepatic bile duct (cHBT) toxicity for primary and secondary liver tumors are based solely on evidence from retrospective observational studies [21]. However, grade ≥ 3 toxicities in up to 22% reflect the need for specific dose constraints to prevent this toxicity [19, 20]. Therefore, we included recommendations for liver lesions in which the PTV is close to or overlaps with the cHBT to enhance awareness for this type of toxicity with major clinical implications [19, 20]. However, it is important to emphasize that this refers only to situations where the GTV itself does not directly affect the cHBT.

#### Pancreas

SBRT in pancreatic ductal adenocarcinoma (PDAC) is typically a balancing act between tumor control and OAR protection: pancreatic head tumors account for 75% of PDAC and the head is enclosed by the radiation-sensitive stomach and duodenum. For this reason, early reports on SBRT in PDAC, typically employing 1–3 fractions, resulted in unacceptably high rates of toxicity [15, 29, 79, 80]. A breakthrough setting the standard for SBRT was a multi-institutional phase II study using five fractions of 6.6 Gy to PTV D_near_min_ prescribed to the 67% isodose with a GTV D_50%_ of 46 Gy, which was well tolerated and achieved 78% local control at 1 year [81]. Since then, fraction numbers ≥ 5 (e.g., 8, 12, 15) were unequivocally recommended to further reduce the risk of late gastrointestinal toxicities [79, 82, 83].

A second strategy for safe SBRT of PDAC became the philosophy to intentionally spare hollow OARs to stay within clinically accepted dose limits whilst accepting underdosing subvolumes of the PTV that overlap or are in close contact with OARs [19, 20, 79]. This includes the concept of SIP, as described above and in more detail elsewhere [41, 42, 84]. Lastly, optimal image-guidance strategies during treatment are of high importance in the context of accurate multiparametric dose prescription delivery and should include oral contrast protocols for each fraction and consideration of real-time MRI [79, 85].

#### Kidney

Over the past years, a significant amount of evidence for SBRT in primary renal cell cancer has accumulated to demonstrate excellent local control and low toxicity rates [15, 24, 86–88]. Although current guidelines are still contradictory in terms of the recommendation for SBRT in kidney cancer, this group of experts recommends the adoption of the practice guideline of the International Society of Stereotactic Radiosurgery recommending SBRT for medically and/or technically inoperable patients and those who are at high risk of postoperative terminal renal insufficiency. [89–91]. Current practical guidelines for kidney SBRT provide the following recommendations: (i) the optimal fractionation should be 25–26 Gy in one fraction or 42–48 Gy in three fractions for larger tumors (level of evidence [LoE] IV), (ii) routine post-SBRT biopsy is not recommended given the absent predictive value regarding oncological outcomes (LoE IIb), (iii) SBRT for primary RCC in a solitary kidney is safe and effective (LoE IIIa), and (iv) cross-axial imaging of the abdomen and surveillance of the chest should be performed every 6 months (LoE IIb) [91]. However, the radiation dose and fractionation for SBRT of RCC vary among different treatment centers. Even though higher local tumor control rates were observed after single-dose SBRT compared with multifraction SBRT in the meta-analysis of Siva and colleagues [24], there is no clear recommendation favoring single-dose SBRT, as further trials in this context are necessary. In the TROG 15.03 FASTRACK II trial, participants either received a single fraction of 26 Gy for tumors that were smaller than 4 cm in maximum diameter, or 42 Gy in 3 fractions for larger tumors [92]. First results of the FASTRACK II trial showed very favorable results (local control and cancer cancer-specific survival of 100%, with grade 3 treatment-related toxicities of 10%) [93]. In this trial, “the investigational treatment will be prescribed to the covering isodose, ensuring that 99% of the PTV is covered by 100% of the dose (D99_PTV_ = 100%)” [93]. However, the authors also state that in cases where OAR dose constraints cannot be respected whilst achieving this level of coverage, an alternative prescription coverage of D95_PTV_ = 100% is acceptable. The authors further recommend a peak dose (D_max_) of ideally 125%, which would result in a normalized equivalent covering isodose of 80%. The acceptable isodose at the periphery should be between 70 and 80%, and the peak dose (i.e. D_max_) should not exceed 143% of the prescribed D_near-min_ [94].

As a matter of principle, the assessment of split renal function measured by, e.g., technetium (99 m) MAG‑3 renal scintigraphy is recommended to determine the relative contribution of excretion for each kidney as well as regional inhomogeneities at baseline [15, 92, 95]. In cases in which the RCC-affected kidney contributes a significantly larger part of the overall excretion function, more conservative dose prescription regimes should be applied.

#### Adrenal gland

For SBRT of adrenal gland metastases, we find insufficient data on normal tissue tolerances in the literature concerning the organ itself. Surgical series indicate that at least 15–30% of healthy adrenal tissue might be sufficient to avoid adrenal insufficiency after bilateral adrenalectomy [27]. This can be achieved in the setting of unilateral SBRT and an intact contralateral adrenal gland. However, SBRT series that included bilateral irradiation or SBRT in the setting of a unilaterally functional adrenal gland indicate an increased risk of adrenal insufficiency of up to 80% in closely monitored patients [96]. Although larger retrospective series indicated a lower risk of clinically apparent adrenal insufficiency [97], cases of insufficiency were also observed in unilaterally treated patients, possibly due to concurrent systemic therapy or insufficient function of the contralateral adrenal gland at baseline.

We therefore recommend endocrine monitoring and follow-up in case of bilateral treatment or in case of unilateral treatment in the setting of a single adrenal gland. Patients who undergo unilateral treatment are at a lower risk of adrenal insufficiency and baseline screening in the planning process might be sufficient if combined with patient education on signs and symptoms of primary adrenal insufficiency. Dose prescription for adrenal metastases should be restricted by dose constraints to the surrounding organs, including small bowel, stomach, kidneys, and liver [19, 20, 79]. Due to the uncertainty regarding the doses required to achieve sufficient local control in different clinical situations, no general recommendation can be made on minimal PTV doses. Large observational studies indicated that dose prescriptions of 50 Gy in 10 fractions or 37.5 Gy in 3 fractions might be associated with improved outcomes compared to lower doses (cut-point: BED 73.2 Gy [26]). Due to these uncertainties, tolerance doses of surrounding OARs should be prioritized over dose escalation.

### Limitations

The following limitations should be taken into account: the expert panel was composed of radiation oncologists and medical physicists from Germany, Austria, and Switzerland, which might limit the generalizability of the recommendations to other regions with different clinical practices or patient populations. Experts were selected based on their involvement in DEGRO/DGMP working groups, publication records, and academic collaborations. While this ensures a high level of expertise, it may also introduce selection bias, favoring opinions and practices prevalent within these specific networks. The systematic literature review was constrained by the scarcity of studies conforming to ICRU report 91, leading to a reliance on literature published within the working groups and other selected renowned groups. This approach might overlook relevant findings from broader sources and potentially bias the underpinning evidence towards the groups’ prevailing views. The use of a modified Delphi method, including online questionnaires and iterative rounds of voting, is a strength in achieving consensus. However, the removal of the abstention option in the second round could force participants to make a definitive choice even if they might still feel insufficiently informed or confident to decide, potentially skewing the consensus. The establishment of predefined thresholds for consensus (high, intermediate, and poor agreement) is standard in Delphi studies. However, the interpretation of these levels in the context of decision making for clinical practice should be approached with caution, especially for statements where consensus was not overwhelming. While the Delphi process can identify areas of agreement among experts, translating these consensus statements into clinical practice requires careful consideration of individual patient circumstances, technological capabilities, and other practical factors not fully captured in the consensus process.

## Conclusion

This Delphi consensus aimed to highlight SBRT dose prescription statements in the light of ICRU report 91. By integrating existing evidence with expert opinions from the DEGRO/DGMP Working Group Stereotactic Radiotherapy and Radiosurgery, our aim was to harmonize clinical practice and clinical trial design. While acknowledging its limitations, this initiative is an important step toward intra-institutional and multicenter consistency to facilitate clinical trials and, ultimately, a better understanding of dosimetric causes of efficacy and safety of SBRT. Mastering multiparametric SBRT dose prescription is right at the interface between physicians and medical physicists, necessitating both professions to be experts sharing a standardized approach. The efforts will serve as a crucial starting point for generating additional evidence, thereby addressing the substantial knowledge gaps that currently exist in this domain.

### Supplementary Information

Figure 1: Exponential rise of published papers on stereotactic radiotherapy from 1990 to 2020 as evidenced by a PubMed search for the term “stereotactic radiotherapy” (performed on 09.04.2024)

Table 1: Delphi process for overaching statements, round 1; statistics.

Table 2: Delphi process for organ-specific statements, round 1; statistics.
