# Supplementary material for: Dose prescription for stereotactic body radiotherapy: general and organ-specific consensus statement from the DEGRO/DGMP Working Group Stereotactic Radiotherapy and Radiosurgery
Source: Strahlenther Onkol. 2024 Jul 12;200(9):737–50. doi: 10.1007/s00066-024-02254-2 (PMC11343978; doi:10.1007/s00066-024-02254-2)

Suppl. Figure 1: Exponential rise of published papers on ‘stereotactic radiotherapy’ from 1990 to 2020 as evidenced by a PubMed search for the term ‘stereotactic radiotherapy‘ (performed on 09.04.2024)

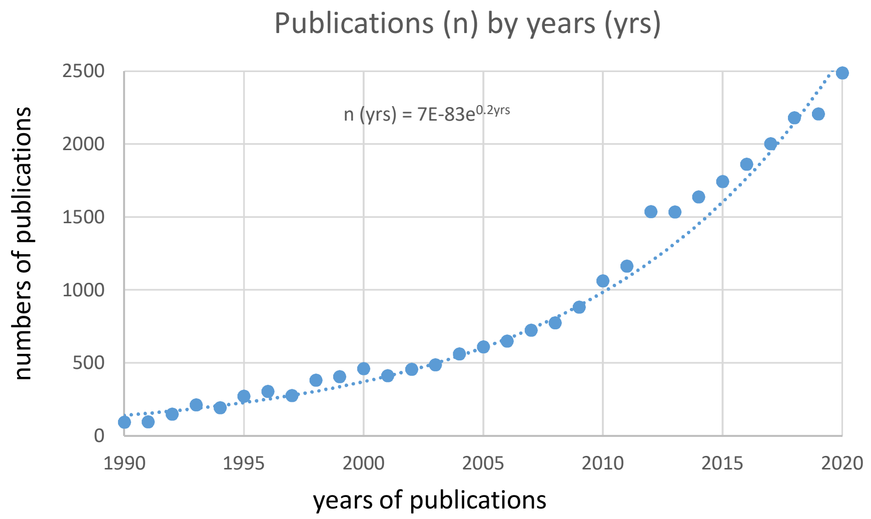

Supplement: Supplementary file 1 — Figure 1: Exponential rise of published papers on stereotactic radiotherapy from 1990 to 2020 as evidenced by a PubMed search for the term “stereotactic radiotherapy” (performed on 09.04.2024) [file 66_2024_2254_MOESM1_ESM.pdf]
