# Supplementary material for: Dose prescription for stereotactic body radiotherapy: general and organ-specific consensus statement from the DEGRO/DGMP Working Group Stereotactic Radiotherapy and Radiosurgery
Source: Strahlenther Onkol. 2024 Jul 12;200(9):737–50. doi: 10.1007/s00066-024-02254-2 (PMC11343978; doi:10.1007/s00066-024-02254-2)
Supplement: Supplementary file 2 — Table 1: Delphi process for overaching statements, round 1; statistics. [file 66_2024_2254_MOESM2_ESM.pdf]

Suppl. Tbl. 1: Delphi process for organ specific statements, round 1, statistics.

Topic	yes	no	abstention
**General requirements – Statement 1**For stereotactic radiotherapy treatments, techn	100	0	0
**Dose prescription – Statement 1**The dose for stereotactic radiotherapy treatments w	88.9	11.1	0
**Dose prescription – Statement 2**The desired dose prescription based on a PTV enc	100	0	0
**Dose prescription – Statement 3**For treatment plan harmonization (e.g., within clinic	88.9	5.55	5.55
**Dose prescription – Statement 5**To adapt dose prescription to specific clinical param	66.7	16.7	16.7
**Beam Technique Planning – Statement 1**Best practice guidelines including simultar	94.4	0	5.6
**Beam Technique Planning– Statement 2**The dose in-homogeneity in the PTV and tl	100	0	0
**Beam Technique Planning– Statement 3**•To create robust SBRT treatment plans, th	88.9	0	11.1
**Beam Technique Planning– Statement 4**For **local Re-SBRT** after prior SBRT, dos	66.7	16.7	16.7
**Dose Calculation - Statement 1**Density override in the treatment planning systems	77.8	11.1	11.1
**Dose Calculation - Statement 2**In **areas with large density inhomogeneities**, the	94.4	0	5.6
**Dose Calculation - Statement 3**The maximum grid size for dose calculation for ster	100	0	0
**Documentation – Statement 1**The dosimetric plan information of stereotactic radioth	83.3	16.7	0
**Documentation – Statement 2**Due to the potential of significant dose reductions from	94.4	5.6	0
