# Supplementary material for: Dose prescription for stereotactic body radiotherapy: general and organ-specific consensus statement from the DEGRO/DGMP Working Group Stereotactic Radiotherapy and Radiosurgery
Source: Strahlenther Onkol. 2024 Jul 12;200(9):737–50. doi: 10.1007/s00066-024-02254-2 (PMC11343978; doi:10.1007/s00066-024-02254-2)
Supplement: Supplementary file 3 — Table 2: Delphi process for organ-specific statements, round 1; statistics. [file 66_2024_2254_MOESM3_ESM.pdf]

Suppl. Tbl. 2: Delphi process for organ specific statements, round 1, statistics.

Topic	Yes	No	Abstention
**Lung - Introduction** Treatment concepts and dose prescription differ depending on tumor location (peripheral,	y	n	a
**Lung - Statement 1** For peripheral tumors dose should be escalated to the GTV/ITV while ensuring a reasonab	92.9	7.1	0
**Lung - Statement 2** For ultracentral tumors, dose sparing of bronchial tree, trachea and esophagus based on w	100	0	0
**Lung - Statement 3** Dose prescription of primary lung tumors should not be different than for pulmonary metast	57.1	28.6	14.3
**Upper abdominal - Statement 1** Patients should be administered prophylactic gastric acid reduction with protor	71.4	0	28.6
**Liver - Statement 1** Dose constraints of patent central bile ducts should have priority over target volume dose p	50	14.3	35.7
**Liver - Statement 2** Liver function must be taken into account for dose prescription especially in HCC (protectec	92.9	0	7.1
**Hepatic metastasis - Statement 1** Liver metastases from colorectal cancer should be treated with higher presc	57.1	28.6	14.3
**Hepatic metastasis - Statement 2** Liver metastases should be treated with higher prescription doses after cher	42.9	35.7	21.4
**Hepatocellular carcinoma - Statement 1** In the absence of a clear dose-response relationship (tumor control p	71.4	7.2	21.4
**Hepatocellular carcinoma - Statement 2** Patients with Child-Pugh scores >8 and with active HBV infection shc	57.1	21.4	21.4
**Cholangiocarcinoma - Statement 1** Dose prescription for primary cholangiocarcinoma should not be different t	78.6	7.1	14.3
**Pancreas - Statement 1** Pancreatic lesions should be treated with 5 or more fractions outside of prospective cli	85.7	7.2	7.1
**Pancreas - Statement 2** For pancreatic lesions, dose sparing of hollow OAR based on well known dose limitatic	100	0	0
**Renal cell cancer - Statement 1** Renal cell cancer should be treated with single fraction SBRT due to radiobiolc	0	80	20
**Renal cell cancer - Statement 2** Prior to SBRT, a split renal function should be performed as determination of	85.7	0	14.3
**Adrenal metastases - Statement 1** Dose prescription for adrenal metastases should prioritize median dose (G	69.2	23.1	7.7

	##########	##########	##########	##########	##########	##########	##########	##########	##########	##########
